# Diabetes and risk of hospitalization due to infection in northeastern Thailand: Retrospective cohort study using population-based healthcare service data

**DOI:** 10.1111/dme.15378

**Published:** 2024-06-09

**Authors:** Pimnara Peerawaranun, Wirichada Pan-ngum, Viriya Hantrakun, Sarah H. Wild, Susanna Dunachie, Parinya Chamnan

**Affiliations:** 1 Mahidol-Oxford Tropical Medicine Research Unit, Faculty of Tropical Medicine, Mahidol University, Bangkok, Thailand; 2 Department of Tropical Hygiene, Faculty of Tropical Medicine, Mahidol University, Bangkok, 10400, Thailand; 3 Usher Institute, University of Edinburgh, Edinburgh, United Kingdom; United Kingdom; 4 Oxford NIHR Biomedical Research Centre, Oxford University Hospitals NHS Foundation Trust, Oxford, United Kingdom; 5 NDM Centre For Global Health Research, Nuffield Department of Clinical Medicine, University of Oxford, Oxford, United Kingdom; 6 Cardiometabolic Research Group, Department of Social Medicine, Sunprasitthiprasong Regional Hospital, Ubonratchathani, Thailand; 7 College of Medicine and Public Health, Ubonratchathani University, Ubonratchathani, Thailand

**Keywords:** Diabetes mellitus, Complications, Infection, risk, hospitalisation

## Abstract

**Background:**

Population-based studies describing the association between diabetes and increased risk of infection have largely been based in high-income countries. There is limited information describing the burden of infectious disease attributable to diabetes in low and middle-income countries.This study aimed to describe the burden and risk of infectious disease hospitalisation in people with diabetes compared to those without diabetes in northeastern Thailand.

**Methods:**

In a retrospective cohort study using electronic health record data for 2012-2018 for 3.8 million people aged ≥20 years in northeastern Thailand, hospitalization rates for any infectious diseases (ICD-10 codes A00-B99) were estimated and negative binomial regression used to estimate rate ratios (RR) for the association between diabetes and infectious disease hospitalization adjusted for age, sex and area of residence.

**Results:**

164,177 people had a diagnosis of diabetes mellitus at any point over the study period. Infectious disease hospitalization rates per 1,000 person-years (95%CI) were 71.8 (70.9, 72.8), 27.7 (27.1, 28.3) and 7.5 (7.5, 7.5) for people with prevalent diabetes, incident diabetes and those without diabetes respectively. Diabetes was associated with a 4.6-fold higher risk of infectious disease hospitalization (RR (95%CI) 4.59 (4.52, 4.66)). RRs for infectious disease hospitalization were 3.38 (3.29, 3.47) for people with diabetes managed by lifestyle alone and 5.29 (5.20, 5.39) for people receiving prescriptions for diabetes drugs.

**Conclusions:**

In this Thai population, diabetes was associated with substantially increased risk of hospitalisation due to infectious diseases and people with diabetes who were on pharmacological treatment had a higher risk than those receiving lifestyle modification advice alone.

**Novelty statement:**

**What is already known?**

- Diabetes was associated with an increased risk of serious infection as defined by need for hospitalization, but the evidence is largely from high-income countries.
- Evidence to describe the burden and risk of infectious disease attributable to diabetes in low and middle-income countries is scarce.

**What has this study found?**

- Infectious disease hospitalization rates in people with and without diabetes were lower than that of high income countries
- Diabtetes was associated with a 4.6-time increased risk of hospitalization due to infection, the magnitude of association being higher than previously reported.
- Risk of infection-related hospitalization was differential by diabetes treatment, with people with diabetes receiving prescriptions for diabetes drugs having a considerably higher risk than those managed by lifestyle alone.

**What are the implications of this study?**

- People with diabetes, particularly those receiving pharmacological treatment, are at high risk of serious infection and further actions are needed to identify factors underlying the excess risk of infection due to diabetes and its treatment to inform appropriate preventive strategies

## Introduction

Diabetes is an important public heath problem worldwide, with an estimated 537 million adults currently living with diabetes of whom over three-quarters are in low- and middle-income countries (LMIC).[1] Diabetes management requires control of multiple risk factors to reduce risks of both micro- and macrovascular complications and mortality.[2, 3]

It is widely accepted that people with diabetes are at increased risk of infection and poor outcomes, particularly for serious infectious diseases, such as pneumonia, cellulitis and infection of bone and subcutaneous tissues, urinary tract infection, diabetic foot disease, surgical site infections and sepsis.[4–10] The increased risk of infection associated with diabetes may be explained by a range of mechanisms, from general and systemic effects of diabetes itself, including hyperglycaemia, impaired physical barrier of skin, altered composition of the gut microbiome, and impaired function of different immune cells, including macrophages, T cells and neutrophils.[11–13] However, few population-based studies have systematically investigated the association between diabetes and risk of infection. In a large registry-based Dutch cohort, type 2 diabetes was associated with a 49% increased risk of hospital-treated infection over a median follow-up of 2.8 years.[5] A matched cohort study using primary care data in England showed that type 2 diabetes almost doubled the risk of infection-related hospitalizations.[14] Increased risk has also been observed for people with type 1 diabetes.[4, 14] However, these studies were from high-income countries and follow-up periods were relatively short. There is limited evidence to describe the burden and risk of infectious diseases attributable to diabetes in LMICs, where diabetes management and burden of infectious diseases differs from that of high income countries.[11, 15]

We aimed to examine rates of hospitalization for any infection and risk of infection-related hospital admssions associated with diabetes relative to people without diabetes in the northeastern region of Thailand using routine healthcare service data.

## Methods

This was a retrospective cohort study based on healthcare service data in men and women aged 20 years and above residing in five provinces in Health Region 10, which is located in the lower part of northeastern Thailand during 2012-2018. Health Region 10 is comprised of five provinces (Srisaket, Umnat Chareon, Mukdahan, Yasothorn and Ubonratchathani) and had a total population of approximately 4.8 million in 2022. People who died before 2012 and those with missing data on age, sex and diabetes status and mortality were excluded. Health Region 10 started implementing systematic screening for diabetes in 2012. All men and women aged ≥40 years and those at high risk (Individuals with hypertension, obesity, and/or a family history of diabetes) based on primary care office’s health records and disease registry data are invited by primary care healthcare workers to undertake screening for diabetes. Fasting blood sugar testing is performed using a point-of-care glucose meter and those with abnormal blood sugar test results are invited for repeat testing. Those with a blood glucose of ≥126 mg% (7 mmol/L) twice are referred to the hospital to confirm diabetes diagnosis, using venous blood or plasma glucose or HbA1c, with diagnostic thresholds aligned to national and international authorities’ recommendations. For newly detected cases, diabetes diagnosis is recorded in both primary care and hospital electronic health records.

Health service data in Thailand are standardized and stored in electronic health records for both outpatient and inpatient care in all levels of healthcare services, primary to tertiary care, although different programmes and software are used. Personal and medical history, physical examination, diagnoses and treatments, health promotion and disease prevention activities are entered and stored in electronic health records in 43 standard folders, all of which are linked with unique national identification numbers.[16] The data are collated in a government centralized database system called ‘Health Data Center’ and used by the National Health Security Office (NHSO) for reimbursement purposes. All Thai people have to register in one of primary care catchment areas in the province in which they live, and generally receive treatment in the healthcare services in their province. People are able to receive treatments and care outside their registered district and province only if they are referred by their doctors. All hospital admissions nationwide are recorded and identified through the NHSO database. All hospital diagnoses are routinely checked and coded by trained coders and then validated by the NHSO. Vital status and, where appropriate, date of death are also included in the database.

Demographic data, diagnoses of diabetes and infectious diseases, treatments and vital status for 3,816,243 people aged 21 years and above living in Health Region 10 were retrieved from the NHSO database and anonymized for this analysis. Data on diabetes diagnosis and treatment were obtained from outpatient and inpatient visits between 2012 and 2018. Diabetes was defined on the basis of presence of the 10^th^ revision of the International Classification of Diseases (ICD-10) codes E10-E11 in outpatient or inpatient records. At least two consecutive records of physician diagnosis were needed to confirm diabetes diagnosis. Data on prescription of diabetes medications including oral hypoglycemic drugs and insulin according to the national drug codes were collected. Data on hospitalizations for any infectious diseases (identified from ICD-10 codes A00-B99) with admission dates were used to identify the outcome.

### Statistical analyses

Characteristics of the study population were described using number (percent) for categorical variables and median (interquartile range, IQR) for continuous variables and comparison of these characteristics between people with and without diabetes and between those with and without hospitalization due to infections were performed using chi-square and Mann-Whitney-U tests for categorical and continuous variables respectively. Incidence rates, overall and by diabetes status, of hospitalization for any infectious diseases were estimated with time at risk in years defined from 2012 (the earliest year for which data are available). This was estimated for both people with prevalent and incident diabetes. Prevalent diabetes was defined as people with a diagnostic code of diabetes in 2012 or before and incident diabetes as those with diabetes diagnostic code newly recorded in 2013-2017. Outcomes (hospitalization due to infection) were identified up to the end of 2018 and infection hospitalization rates and 95% confidence intervals (CIs) estimated. Negative binomial regression analyses were performed to assess the associations between diabetes and risk of hospitalisation due to infections. Negative binomial regression modelling allowed for examining the temporal relationship between an exposure of interest, in this case having diabetes, in a particular year and hospitalizations in the following years. Two multivariable regression models were developed: Model A with diabetes and Model B with diabetes treatment being a main exposure as compared to no diabetes, adjusted for other participant characteristics. Rate ratios (RR) with corresponding 95% CIs were reported. A 2-tailed P value of 0.05 was considered statistically significant. All analyses were conducted using Stata version 16.0.

## Results

From a total of 3,816,243 people aged 21 years and above residing in five provinces of Health Region 10, 3,795,856 (99.5%) had complete data on age, sex, residential areas, diabetes status and mortality and they were tracked for diabetes diagnosis, and history and causes of hospitalization during 2012-2018. The cohort included 164,177 people who had a diagnosis of diabetes mellitus at any point over the study period (2012-2017). Among those with diabetes, 133,117 (80.1%) received at least one prescription for a diabetes drug and the remainder were managed with lifestyle modification alone.

Table 1 shows characteristics of the study population, overall and stratified by diabetes. The median age (Interquartile range, IQR) of the whole population was 46 (34 – 58) years, with approximately 22% being 60 years and older People with diabetes were older than those without diabetes and there was a larger proportion of women in the population with diabetes (62%) than the population without diabetes (50%). People with and without diabetes were comparable in terms residental area distribution. Characteristics of the study population stratified by hospitalization due to any infectious diseases are described in Table 2. Over the study period of 6 years (2013-2018), 169,451 (4.5%) people had at least one hospitalization due to infection. People with at least one infection disease hospitalization during the study period were older and more likely to be female than those without. Those with infections requiring hospitalization were more likely to have diabetes than those without.

### Hospital admissions due to infections

Table 3 describes the overall and annual number and rates of hospitalization due to any infections in people with and without diabetes. Annual proportions of the population admitted to hospital for infectious diseases ranged from 2.3% to 4.2% in people with diabetes and from 0.7 to 0.9% in those without diabetes. Those with diabetes had a total of 33,035 hospital admissions due to any infection between 2013 and 2018 (Overall rate of hospital admissions with an infection of 49.2 per 1,000 person-years), while those without diabetes had 163,566 hospital admissions over the same period (7.5 per 1,000 person-years). Hospitalization rates with infections for people with prevalent diabetes at the start of 2013 were higher than for people whose diabetes was diagnosed between 2013 and 2017 (71.8 and 27.7 per 1,000 person-years respectively). In subgroup analyses infection hospitalization rates were similar for those with incident diabetes dignosed in different years between 2013 and 2017, ranging from 26.2 to 28.5 per 1,000 person-years.

### Association between diabetes mellitus and risk of hospitalization due to infections

Table 4 shows the association between diabetes status and risk of hospitalization due to any infections in the residents of Health Region 10. Age, sex, province of residence and diabetes were independently associated with the risk of hospital admission due to infections in multivariable negative binomial regression models. Diabetes was associated with a 4.6-fold increase in risk of infections requiring hospitalization after controlling for age, sex, and province of residence (Model A). When stratified by diabetes treatment, patients with diabetes who received lifestyle modification advice alone and those who received both diabetes drug prescription and lifestyle modification advice had a 3.4- and 5.3-fold higher risk of hospitalization than people without diabetes (Model B). Risk of hospitalization due to infection increased with age, (Adjusted IRR of 1.17, 2.46 and 3.89 for those aged 41-60, 61-80 and ≥ 81 years as compared to those aged 20-40 years). Compared to males, females had a 3% lower risk of infection related hospitalization. Risk of hospitalization due to infection varied by province, with people from Sisaket having the highest risk.

## Discussion

In this large contemporary cohort of the population in Northeastern Thailand, a large number of hospitalizations due to infectious diseases occurred over the study period with the infection hospitalization rates being substantially higher in people with diabetes than those without. Diabetes was associated with almost 5-fold higher risk of hospital admissions due to infection after adjusting for age, sex and province. Patients who received both diabetes drug prescription and lifestyle modification advice had a higher risk of infection related hospitalization than those who received lifestyle modification advice alone.

Hospital admissions due to infection vary considerably across age groups, populations and healthcare settings. While evidence from LMICs is scarce, rates of hospitalization due to infection in our study were lower than the figures reported in high-income countries (0.7-0.9% vs.1.3-5.0% per year, respectively).[5, 17, 18] In addition to differences in sanitation, immunization, public health between countries by income, differences in the definitions and methods to ascertain infectious diseases and age structure of the population existed between studies. Besides, it is possible that the difference in hospitalization rates may at least in part be attributable to differential infectious disease patterns and clinical thresholds for hospitalisation which vary by countries, resources and practice. Consequently, comparison across studies is difficult and future research using standard definitions and ascertainment methods of infectious diseases and age-standardized estimates are needed to make comparisons within and between countries.

Variation in the prevalence of diabetes across provinces was observed in our study. This is consistent with results from the National Heath Examination Survey showing significant discrepancies in diabetes prevalence across Thailand’s geographic regions.[19] The differences in diabetes prevalence may be explained by several potential factors including the different starting times of systematic screening, differential implementation strategies across the health provincial sectors, and differences in population demographics, socio-economic status and behaviour across provinces.

People with diabetes are at increased risk of serious infectious disease as indicated by a need for hospital admission. While most studies examined risk of certain types of infection based on clinic- or hospital clinic settings,[4, 6, 20, 21] evidence from population-based studies on infection risk due to diabetes is limited and inconsistent. A small matched-pair cohort in Australia suggested that, although the number of infection episodes differed between those with and without diabetes, diabetes was not associated with risk of having at least one infection over follow-up period.[9] A large prospective cohort in the Danish population suggested that type 2 diabetes was associated with a 49% increased risk of hospital-treated infections,[5] while our study found that diabetes increased the risk of hospital admission more than four-fold. In the Atherosclerosis Risk in Communities (ARIC) study conducted in the United States, 12,379 participants were followed-up for a median of 23.8 years, during which there were 4,229 new hospitalisations for infection, and diabetes was associated with an almost 2-fold and 6-fold increase in risk of hospitalization for any infection and foot infection respectively.[10] The difference in the risk of infection related to diabetes between studies may partly be explained by the different definition of outcomes between studies. While the Australian study used self-reported infection and the Danish study defined the outcome as diagnoses of any infection captured from inpatient and outpatient hospital records, our study was limited to hospital admissions with infection, similar to the US ARIC study. This heterogeneity suggests differences between populations including underlying risk of infection but the higher risk associated with diabetes in all studies underlines the clinical importance of diabetes for prevention and management of infectious diseases in all populations.

Patterns of infectious diseases (causative agents and sites of infection) may also be different across countries and world regions. A large epidemiologic study in northeastern Thailand suggests that up to two-thirds of community-acquired bacteremia were caused by gram negative bacteria and the most common site was lower respiratory tract infection [22] Further, compelling evidence in LMICs suggests that diabetes increases susceptibility and severity of certain types of infectious diseases, including Mycobacterium tuberculosis, Burkholderia pseudomallei, E. coli and Klebsiella as well as Staphylococcus aureus, Streptococcal species and Influenza.[11] We were unable to investigate into this as the dataset available for our analysis did not include coding for individual infectious diseases and data on sites of infection.”

Heterogeneity among people with diabetes may influence risk of infectious disease and subsequent hospitalization. Our study supports this by showing a higher risk of infection requiring hospital admission in those receiving diabetes drug prescriptions than those receiving lifestyle modification advice only for diabetes management. We did not have data on duration of diabetes among the prevalent cohort but identified higher risk for infection related hospitalization for people with prevalent diabetes than for people with incident diabetes. A further population-based cohort study using linked medical databases in Denmark suggested that risk of hospital-treated infection differed between choice of initial glucose-lowering drugs, with those starting pharmacological treatment with insulin or sulfonylureas having higher risk of hospital-treated infection than those starting treatment with metformin.[23] Increased risk of hospital-treated infection associated with prevalent diabetes and needs for diabetes medication suggest that this group of patients may have a longer diabetes duration or be in the later stage of the disease with more co-morbidities, hence a higher risk of infection. However, due to data unavailability, factors that may reflect an individual’s immune status and hence vulnerability to serious infectious diseases were not accounted for in our study. These include health behaviours, blood glucose levels, comorbidities, namely cancer and chronic kidney disease, and prescription of corticosteroids, which individually or together could confound the association between diabetes and hospital admission with infection.

People with different types of diabetes may have different risk of serious infection. Although the association of serious infectious diseases with diabetes have been observed, the magnitude of the association appeared to be different for type 1 and type 2 diabetes.[4, 14] As we did not have access to data on specific types of diabetes, we were unable to describe if rates and risk of infection hospitalization differed by types of diabetes. However, according to a large national diabetes management survey, type 2 diabetes represented more than 95% of all diabetes in Thailand, with only 4% and less than 1% being type 1 diabetes and other types of diabetes.[24] Although rates and risk of infection hospitalization for type 2 diabetes may be similar to the figures reported in our study, the results for other types of diabetes in the Thai population might have been different and would require further studies.

This study is among the first to examine infectious disease hospital admission rates and their association with diabetes in a LMIC. Our study was based on data in a contemporary large Thai cohort using standard statistical techniques. However, our study had a number of limitations. First, diagnoses of diabetes and infectious diseases were based on ICD-10 codes from electronic health records and the national reimbursement database and we were not able to obtain laboratory data, particularly for measures of glycemia and microbiology results. This could result in misclassification of diabetes and infectious diseases, which might have altered the association between the two conditions. However, the potential for misclassification is expected to be modest as in the present study at least two consecutive records of physician diagnosis of diabetes were needed to confirm if an individual had diabetes and infectious diseases were ascertained through the linkage to the NHSO database, with all hospital diagnoses verified for reimbursement purposes. Due to data on the exact date of diabetes diagnosis and hence time to event were not available, negative binomial regression represents an appropriate statistical approach for this analysis. It is possible; however, that if data on ‘date of diabetes diagnosis’ and ‘time to diagnosis’ were available, results on infection hospitalization rates and risk associated with diabetes using survival analysis may have been altered. It is also possible that some patients were newly diagnosed with diabetes during the time of hospital admission. No data on whether an individual had diabetes diagnosed during a particular hospital admission were available in our study. This could have altered the association reported in this study. However, with systematic screening in place in Thailand this is likely to affect only a small number of people, and hence the potential for misclassification of diabetes status to bias the association with infectious disease hospitalisation towards the null can be expected to be modest. Further, data used in our analyses did not include private healthcare services, hence underestimation of numbers of people with diabetes and infectious diseases was possible. However, as the large majority of Thai people in provincial areas receive government healthcare services for diabetes as it needs regular follow-up and serious infectious diseases need hospital admission, we believe our dataset captured most of the diagnoses of clinical importance.

In conclusion, diabetes was associated with a substantially increased risk of hospitalization due to infectious diseases and people with diabetes who were on pharmacological treatment had a higher risk than those receving lifestyle modification advice alone. Future research is needed to examine factors underlying the excess risk of infection due to diabetes and the association with certain types of infectious diseases in order to inform appropriate preventive strategies.

## Figures and Tables

**Table 1:** Characteristics of people with and without diabetes registered to receive health care in Health Region 10 of north-eastern Thailand between 2012-2017

Characterictics	Total (3,795,856)	People with diabetes (164,177)	People without diabetes (3,631,679)	P-value*
Median age in years (IQR)	46 (34-58)	62 (54-71)	45 (33?57)	<0.001
Age groups, n (%)				<0.001
21 – 40 years	1,465,549 (39)	5,883 (4)	1,459,666 (40)	
41 – 60 years	1,505,282 (40)	67,384 (41)	1,437,898 (40)	
61 – 80 years	672,204 (18)	78,933 (48)	593,271 (16)	
≥ 81	140,751 (4)	11,965 (7)	128,786 (4)	
Sex, n (%)				<0.001
Male	1,883,488 (50)	62,211 (38)	1,821,277 (50)	
Female	1,912,368 (50)	101,966 (62)	1,810,402 (50)	
Province, n (%)				<0.001
Sisaket	1,229,577 (32)	42,549 (26)	1,187,028 (33)	
Ubon Ratchathani	1,508,327 (40)	73,618 (45)	1,434,709 (40)	
Yasothorn	455,980 (12)	20,960 (13)	435,020 (12)	
Amnat Charoen	314,769 (8)	15,645 (10)	299,124 (8)	
Mukdahan	287,203 (8)	11,405 (7)	275,798 (8)	

* P-value for comparison between groups by diabetes status using chi-square and Mann-Whitney-U tests for categorical and continuous variables respectively

**Table 2:** Characteristics of participants with and without hospitalizations due to infection during 2013-2018

Characteristics	Total (N= 3,795,856)	No hospital admission with infection (N= 3,626,405)	Hospital admission with infection (N= 169,451)	P-value*
Age in years, median (IQR)	46 (34 – 58)	45 (34 – 58)	57 (40 – 71)	<0.001
Age groups, n (%)				<0.001
21 – 40 years	1,465,549 (39)	1,423,010 (39)	42,539 (25)	
41 – 60 years	1,505,282 (40)	1,451,849 (40)	53,433 (32)	
61 – 80 years	672,204 (18)	615,564 (17)	56,640 (33)	
≥ 81	140,751 (4)	123,926 (3)	16,825 (10)	
Sex, n (%)				<0.001
Male	1,883,488 (50)	1,801,773 (50)	81,715 (48)	
Female	1,912,368 (50)	1,824,632 (50)	87,736 (52)	
Province, n (%)				<0.001
Sisaket	1,229,577 (32)	1,168,287 (32)	61,290 (36)	
Ubon Ratchathani	1,508,327 (40)	1,447,014 (40)	61,313 (36)	
Yasothorn	455,980 (12)	435,537 (12)	20,443 (12)	
Amnat Charoen	314,769 (8)	301,180 (8)	13,589 (8)	
Mukdahan	287,203 (8)	274,387 (8)	12,816 (8)	
Diabetes during 2012-2017, n (%)				<0.001
Yes	164,177 (4)	137,615 (4)	26,562 (16)	
No	3,631,679 (96)	3,488,790 (96)	142,889 (84)	

* P-value for comparison between groups using chi-square and Mann-Whitney-U tests for categorical and continuous variables respectively

**Table 3:** Number and rates of hospitalization due to any infections in northeastern Thailand between 2013-2018, overall and by diabetes status and year of diabetes diagnosis

	Number	Hospitalization due to infections, n (%)						Incidence rates of infections per 1,000 person-year (95% CI)
		2013	2014	2015	2016	2017	2018	
Total population	3,795,856	34,516/ 3,686,238 (0.9)	30,450/ 3,710,643 (0.7)	34,408/ 3,734,869 (0.9)	32,785/ 3,756,722 (0.9)	31,088/ 3,776,631 (0.8)	33,354/ 3,795,856 (0.9)	8.8 (8.7, 8.8)
Diabetes status								
without DM	3,631,679	30,734 (0.9)	25,822 (0.7)	29,135 (0.8)	26,531 (0.7)	24,835 (0.7)	26,509 (0.7)	7.5 (7.5, 7.5)
with DM	164,177	3,782 (2.3)	4,628 (2.8)	5,273 (3.2)	6,254 (3.8)	6,253 (3.8)	6,845 (4.2)	49.2 (48.7, 49.8)
Type of diabetes cases								
Prevalent cases*	54,559	3,782 (6.9)	3,916 (7.2)	3,921 (7.2)	4,142 (7.6)	3,876 (7.1)	3,881 (7.1)	71.8 (70.9, 72.8)
Incident cases*	109,618	-	712/ 24,405 (2.9)	1,352/ 48,631 (2.8)	2,112/ 70,484 (3.0)	2,377/ 90,393 (2.6)	2,964/ 109,618 (2.7)	27.7 (27.1, 28.3)
Incident DM by diagnosis year								
2013	24,405	-	712 (2.9)	673 (2.8)	730 (3.0)	654 (2.7)	711 (2.9)	28.5 (27.6, 29.5)
2014	24,226	-	-	679 (2.8)	700 (2.9)	640 (2.6)	592 (2.4)	26.9 (25.9, 28.0)
2015	21,853	-	-	-	682 (3.1)	564 (2.6)	594 (2.7)	28.1 (26.8, 29.4)
2016	19,909	-	-	-	-	519 (2.6)	523 (2.6)	26.2 (24.6, 27.8)
2017	19,225	-	-	-	-	-	544 (2.8)	28.3 (26.0, 30.8)

* Prevalent cases = diabetes diagnosed in 2012 and before, Incident cases = diabetes newly diagnosed in 2013-2017

**Table 4:** Rates of infection hospitalization and rate ratios (RR) describing the association between diabetes, diabetes treatment and risk of hospitalization due to any infection during 2012-2018 in the whole population of 3,795,856 people living in Health Region 10 using negative binomial regression

Factors	Number	Rates of infection hospitalizations per 1,000 person-year (95% CI)	Univariate negative binomial model		Multivariable negative binomial model A†		Multivariable negative binomial model B‡	
			Crude RR (95% CI)	p-value	Adjusted RR (95% CI)	p-value	Adjusted RR (95% CI)	p-value
**Diabetes status**								
People without diabetes	3,631,679	7.5 (7.5, 7.5)	1	1	1	1	1	1
People with diabetes	164,177	49.2 (48.7, 49.8)	6.23 (6.13, 6.33)	<0.001	4.59 (4.52, 4.66)	<0.001	-	-
**Diabetes treatment**								
			Reference to no diabetes				Reference to no diabetes	
Lifestyle modification alone	31,060	36.9 (36.1,37.7)	4.75 (4.63, 4.88)	<0.001			3.38 (3.29, 3.47)	<0.001
Both diabetes drugs and lifestyle modification	133,117	56,2 (55.5, 56.9)	7.06 (6.93, 7.19)	<0.001			5.29 (5.20, 5.39)	<0.001
**Age (years)**								
21 – 40	1,465,549	4.6 (4.6, 4.6)	1	1	1	1	1	1
41 – 60	1,505,282	6.0 (5.9, 6.0)	1.30 (1.29, 1.32)	<0.001	1.17 (1.15, 1.18)	<0.001	1.17 (1.15, 1.18)	<0.001
61 – 80	672,204	15.0 (14.9, 15.1)	3.30 (3.26, 3.35)	<0.001	2.46 (2.43, 2.49)	<0.001	2.46 (2.43, 2.49)	<0.001
≥ 81	140,751	21.6 (21.3, 21.9)	4.74 (4.64, 4.83)	<0.001	3.89 (3.82, 3.97)	<0.001	3.92 (3.84, 3.99)	<0.001
**Sex**								
Male	1,883,488	7.2 (7.1, 7.2)	1	1	1	1	1	1
Female	1,912,368	7.9 (7.8, 7.9)	1.10 (1.09, 1.11)	<0.001	0.97 (0.96, 0.98)	<0.001	0.97 (0.96, 0.98)	<0.001
**Province**								
Sisaket	1,229,577	6.8 (6.8, 6.9)	1	1	1	1	1	1
Ubon Ratchathani	1,508,327	8.4 (8.4, 8.5)	1.24 (1.22, 1.25)	<0.001	1.27 (1.26, 1.28)	<0.001	1.27 (1.25, 1.28)	<0.001
Yasothorn	455,980	7.7 (7.6, 7.8)	1.13 (1.11, 1.15)	<0.001	1.10 (1.08, 1.11)	<0.001	1.10 (1.08, 1.11)	<0.001
Amnat Charoen	314,769	7.4 (7.3, 7.5)	1.09 (1.07, 1.11)	<0.001	1.06 (1.04, 1.08)	<0.001	1.06 (1.04, 1.08)	<0.001
Mukdahan	287,203	7.5 (7.3, 7.6)	1.10 (1.07, 1.12)	<0.001	1.13 (1.11, 1.15)	<0.001	1.13 (1.10, 1.15)	<0.001

† Model A describes RR for diabetes compared with no diabetes adjusted for other factors in the table.

‡ Model B describes RR for diabetes with or without diabetes drugs compared with no diabetes adjusted for other factors in the table.

## Data Availability

The datasets generated during and/or analyzed in the current study are available from the corresponding author upon reasonable request.
